# Identification of *Plasmodium* PI4 kinase as target of MMV390048 by chemoproteomics

**DOI:** 10.1186/1475-2875-13-S1-P38

**Published:** 2014-09-22

**Authors:** Sonja Ghidelli-Disse, Maria Jose Lafuente-Monasterio, David Waterson, Michael Witty, Yassir Younis, Tanya Paquet, Leslie J Street, Kelly Chibale, Francisco Javier Gamo-Benito, Marcus Bantscheff, Gerard Drewes

**Affiliations:** 1 Cellzome GmbH - a GSK Company, Heidelberg, Germany; 2 GlaxoSmithKline, Tres Cantos, Spain; 3 Medicines for Malaria Venture, Geneva, Switzerland; 4 University of Cape Town, Cape Town, South Africa

## 

Most antimalarial drugs face decreased efficacy due to the emergence of resistant parasites. Therefore, the discovery of new antimalarial medicines is focused on new drugs that act by novel mechanisms and are active against different *P. falciparum* development stages. Screening of a focused compound library for antiparasitic activity, lead to identification of a novel class of compounds with activity against *P. falciparum*, 2-aminopyridines. The selected hits were validated and subjected to a lead optimization program resulting in the pre-clinical candidate MMV390048. Here we report an unbiased chemoproteomics strategy for the identification of targets of MMV390048. An analogue of MMV390048 containing a primary amine function for immobilization in a permissive position was synthesized and covalently immobilized on sepharose beads. Affinity capturing of potential target proteins from a *P. falciparum* blood stage extract was performed in the absence and presence of an excess of MMV390048 in the extract to delineate target proteins for which capturing is competitively inhibited. All proteins captured by the beads were quantified by isotope tagging of tryptic peptides followed by LC-MS/MS. Notably MMV390048 competitively inhibited the binding of only a single protein, *P. falciparum* PI4 kinase, to the beads. However, the immobilization of a drug compound via a linker may not be compatible with the binding to all of its targets. Therefore, we also performed a capturing experiment with kinobeads, which represent a combination of immobilized promiscuous ATP competitive kinase inhibitors (Bantscheff *et al.*, 2007). As in the previous experiment, PfPI4K was the only *P. falciparum* protein which exhibited a reduction of bead binding upon the addition of MMV390048 to the extract. Knowledge of the target will accelerate the drug discovery process.

